# Ensuring transparency and minimization of methodologic bias in preclinical pain research: PPRECISE considerations

**DOI:** 10.1097/j.pain.0000000000000458

**Published:** 2015-12-16

**Authors:** Nick A. Andrews, Alban Latrémolière, Allan I. Basbaum, Jeffrey S. Mogil, Frank Porreca, Andrew S.C. Rice, Clifford J. Woolf, Gillian L. Currie, Robert H. Dworkin, James C. Eisenach, Scott Evans, Jennifer S. Gewandter, Tony D. Gover, Hermann Handwerker, Wenlong Huang, Smriti Iyengar, Mark P. Jensen, Jeffrey D. Kennedy, Nancy Lee, Jon Levine, Katie Lidster, Ian Machin, Michael P. McDermott, Stephen B. McMahon, Theodore J. Price, Sarah E. Ross, Grégory Scherrer, Rebecca P. Seal, Emily S. Sena, Elizabeth Silva, Laura Stone, Camilla I. Svensson, Dennis C. Turk, Garth Whiteside

**Affiliations:** a Department of Neurobiology, Boston Children's Hospital, Harvard Medical School, Boston, MA, USA; b Department of Anatomy, University of California San Francisco, San Francisco, CA, USA; c Department of Psychology and Alan Edwards Centre for Research on Pain, McGill University, Montreal, Canada; d Department of Pharmacology, College of Medicine, University of Arizona, Tucson, AZ, USA; e Pain Research, Department of Surgery and Cancer, Imperial College, London, United Kingdom; f Centre for Clinical Brain Sciences, University of Edinburgh, Edinburgh, United Kingdom; Departments of g Anesthesiology,; h Neurology, and; i Psychiatry, University of Rochester, Rochester, NY, USA; j Department of Anesthesiology, Wake Forest School of Medicine, Winston-Salem, NC, USA; k Department of Biostatistics, Harvard University, Boston, MA, USA; l Department of Anesthesiology, University of Rochester, Rochester, NY, USA; m Clinical and Rehabilitative Medicine Research Program, United States Army Medical Research and Materiel Command, Fort Detrick, MD, USA; n Department of Physiology and Pathophysiology, Friedrich-Alexander Universität, Erlangen-Nürnberg, Germany; o Institute of Medical Sciences, University of Aberdeen, Aberdeen, United Kingdom; p Eli Lilly & Co., Indianapolis, IN, USA; q Department of Rehabilitation Medicine, University of Washington, Seattle, WA, USA; r Neuroscience Discovery Research, Eli Lilly & Co., Indianapolis, IN, USA; s Policy Department, Wellcome Trust, London, United Kingdom; Departments of t Medicine and; u Oral Surgery, University of California San Francisco, San Francisco, CA, USA; v National Centre for Replacement, Refinement & Reduction of Animals in Research, London, United Kingdom; w Kent, United Kingdom; x Department of Biostatistics and Computational Biology and Department of Neurology, University of Rochester, Rochester, NY, USA; y Neurorestoration group, King's College, London, United Kingdom; z School of Behavioral and Brain Sciences, University of Texas at Dallas, TX, USA; aa Department of Neurobiology, University of Pittsburgh, Pittsburgh, PA, USA; bb Department of Anesthesiology, Perioperative and Pain Medicine, Stanford University School of Medicine, Palo Alto, CA, USA; cc Department of Neurobiology, University of Pittsburgh, Pittsburgh, PA, USA; dd Office of Career and Professional Development, University of California San Francisco, San Francisco, CA, USA; ee Faculty of Dentistry & Alan Edwards Centre for Research on Pain, McGill University, Montreal, Canada; ff Department of Physiology and Pharmacology, Karolinska Institutet, Stockholm, Sweden; gg Department of Anesthesiology and Pain Medicine, University of Washington, Seattle, WA, USA; hh Discovery Research, Purdue Pharma, Cranbury, NJ, USA

**Keywords:** Transparent reporting, Consensus, Bias, Internal validity

## Abstract

There is growing concern about lack of scientific rigor and transparent reporting across many preclinical fields of biological research. Poor experimental design and lack of transparent reporting can result in conscious or unconscious experimental bias, producing results that are not replicable. The Analgesic, Anesthetic, and Addiction Clinical Trial Translations, Innovations, Opportunities, and Networks (ACTTION) public–private partnership with the U.S. Food and Drug Administration sponsored a consensus meeting of the Preclinical Pain Research Consortium for Investigating Safety and Efficacy (PPRECISE) Working Group. International participants from universities, funding agencies, government agencies, industry, and a patient advocacy organization attended. Reduction of publication bias, increasing the ability of others to faithfully repeat experimental methods, and increased transparency of data reporting were specifically discussed. Parameters deemed essential to increase confidence in the published literature were clear, specific reporting of an a priori hypothesis and definition of primary outcome measure. Power calculations and whether measurement of minimal meaningful effect size to determine these should be a core component of the preclinical research effort provoked considerable discussion, with many but not all agreeing. Greater transparency of reporting should be driven by scientists, journal editors, reviewers, and grant funders. The conduct of high-quality science that is fully reported should not preclude novelty and innovation in preclinical pain research, and indeed, any efforts that curtail such innovation would be misguided. We believe that to achieve the goal of finding effective new treatments for patients with pain, the pain field needs to deal with these challenging issues.

## Abstract 2

Improved reporting standards, and adoption of experimental protocols that emphasize control of experimental bias, are likely to improve understanding and confidence in pain biology.

## 1. Introduction

The scientific community is highly regulated and represents a complex interplay of relationships between public and private enterprises. Scientific discoveries relating to the basic biology of pain are primarily disseminated through publication in peer-reviewed journals. These studies inform the field, allowing the generation of hypotheses that may yield novel pain therapeutics. Despite great advances in the treatment of pain, there is a concern that discovery of novel, efficacious therapeutics for pain has slowed. Clinical trial failures raise many questions, including the relevance of preclinical hypotheses and the accuracy of animal models to predict the biology of the human condition. However, because clinical trials are only performed after successful replication at the preclinical level, their failure does not reflect a lack of replicability of the original results, but more likely the complexity of human clinical trials. Indeed, there are many reasons why a compound may fail in clinical settings, such as inability to use effective doses, failure to engage the target at the correct time, unsuitable pharmacokinetics, wrong patient group, large placebo response, insensitive outcome measures, and patient drop out.

As well as the above issues, there is growing concern about the lack of scientific rigor and transparent reporting^8,10^ across many preclinical fields of biological research. Poor experimental design and lack of transparency can result in conscious or unconscious experimental bias^19^ that may result in findings that are not replicable. Concerns have also been raised that similar problems may exist in preclinical pain research.^14,15^ Experimental bias (something that can be mitigated) differs from scientific fraud (something that is virtually impossible to prevent if an individual is determined) in that scientific fraud is the creation of data that were either never generated at all or that were generated by methods other than those described. Experimental bias is unintentional and often a result of poor internal validity, which ultimately prevents the scientist from correctly attributing an observed effect to a particular treatment or intervention. Internal validity is therefore extremely important both to scientists who are looking to generate hypotheses based on reliable data and to scientists seeking to test hypotheses reported in the literature.

Improved uniform reporting standards, and adoption of experimental protocols that emphasize control of experimental bias, are likely to improve understanding and confidence in pain biology. Transparent reporting requires that authors fully describe “what was actually done” and does not per se dictate the experimental approach. Improving standards of reporting will increase the number of articles that can be used for meta-analyses. To address the issues discussed, the Preclinical Pain Research Consortium for Investigating Safety and Efficacy (PPRECISE) Working Group was convened with the goal of identifying important information that should be included in experimental publications. The many factors that influence the outcome of clinical trials have been reviewed^5^ and will not be addressed here. Although this commentary will focus on transparency of reporting and improving experimental design primarily in behavioral experimentation, the general concepts to reduce bias should apply across all scientific experimentation in preclinical pain studies.

## 2. Method

The Analgesic, Anesthetic, and Addiction Clinical Trial Translations, Innovations, Opportunities, and Networks (ACTTION) public–private partnership with the U.S. Food and Drug Administration sponsored a consensus meeting of the PPRECISE Working Group that took place on the 11th and 12th of July 2013. The entire group included international participants from universities, government agencies, industry, and a patient advocacy organization (ie, the authors) and all attendees were present during all sessions on both days. There were no “breakout” groups. Forty participants were selected from the academic and pharmaceutical/biotechnology fields on the basis of their research, clinical, or administrative expertise relevant to the fundamental understanding of neurobiological mechanisms of pain and experimental design and preclinical evaluation of treatments for chronic pain. The organizing committee was Dr A. I. Basbaum, Dr F. Porreca, Dr A. S.C. Rice, and Dr C. J. Woolf, with Dr R. H. Dworkin and Dr D. C. Turk representing ACTTION on an ex officio basis. The committee above chose all the other participants/coauthors, based on their contribution to pain research either as basic scientist, clinical scientist, or industry scientist, or involved in publication or regulation of pain research with an additional emphasis on including representative junior investigators. The meeting was designed to reflect a broad representation of relevant disciplines and perspectives, while limiting the number of attendees to promote productive and efficient discussion. Before the meeting, all attendees received 12 publications from the literature relating to reporting standards in animal studies of pain (Rice et al.^14,15^ and Currie et al.^4^), stroke (Macleod et al.^10^ and Sena et al.^16^), and articles more broadly addressing reporting standards and experimental design across biological research (Kilkenny et al.,^8^ van der Worp et al.,^19^ and Landis et al.^9^). To facilitate discussion, meeting presentations were organized into 3 main sessions: (1) design and reporting of preclinical pain research (from the perspectives of academic and industrial scientists, funders, and regulatory bodies); (2) reporting of preclinical pain research (from an Editor's perspective); and (3) “good laboratory practice” domains: designing, conducting, and reporting preclinical studies of pain—challenges and opportunities (covering aspects of internal and external validities). The format allowed for questions from the floor during the presentations and further time was allocated at the end of each session for further questions from the floor to the speakers as a panel.

The following is based on the background presentations and extensive discussions and debates at the consensus meeting. A draft manuscript was circulated to all authors and after iterative revisions, a consensus was achieved; all authors approved the final version of this article.

## 3. Results

**Figure 1:**
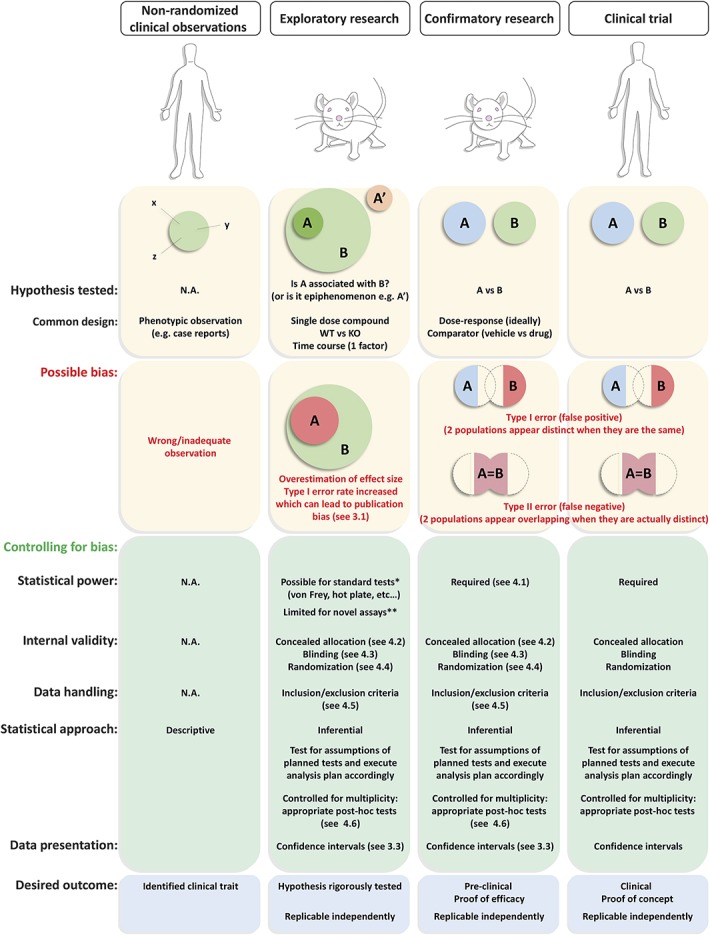
Comparison of different categories of experimental research, illustration of possible bias, and how this may be minimized with best practice to achieve a reliable outcome. **Nonrandomized clinical observations**, which are typically case reports, are effectively phenotypic observations often of individuals. As such, the recommended statistical approach is descriptive (summarizing the data without making further interpretations) rather than inferential (meaning, formal hypothesis testing or estimation). Such studies are most useful for hypothesis generation because inferences are not being made and so bias is not an issue. For **exploratory animal research**, the hypothesis being tested can be represented as “Is a mechanism (A) associated with the disease process (eg, neuropathic pain) (B) or is it an epiphenomenon (A')?”. Type 1 errors occur when the null hypothesis is incorrectly rejected; that is, the result is a “false positive” (shown by an enhanced size of A within B in the lower half of the figure). False positives commonly occur because of experimental bias and are arguably of most concern in exploratory research as it is far more likely that a false positive would be published than a false negative because of publication bias favoring the publication of “positive” findings supporting exciting new hypotheses. We propose that careful attention to internal validity (section 4) can help reduce false positives and increase the rigor by which hypotheses are tested. Clear description of inclusion/exclusion criteria, use of appropriate statistical tests after initial tests for normality, and controlling for multiplicity of statistical testing are all recommended to reduce biasing the importance of a finding. *It is possible to determine the appropriate sample size using power calculations because an effect size can be estimated from the historical data generated using standard endpoints of pain assessment in animals. **It might not be possible to determine the sample size using power calculations when both the mechanism and the endpoint are novel; however, one might want to include a known analgesic or modulator of a known mechanism in the experimental design to better define assay sensitivity. **Confirmatory (preclinical) research** often compares in approach with clinical trials and deals with the question of, for example, whether compound (A) is different from vehicle (B) in much the same way that **clinical trials** compare a test substance with placebo. This type of experiment tests existing hypotheses (eg, whether antagonism of target A results in antihyperalgesia in a particular model of neuropathic pain). We recommend that the use of power calculations to determine the appropriate sample size can and should be performed because the model and the endpoint are typically validated, and effect sizes of standard analgesics in the model and test are known. Dose responses (rather than single-dose studies) should be performed when possible and should be analysed by the appropriate statistics after tests for normality and with post hoc analysis strategies controlled for multiplicity. It is also recommended to include a comparator such as a positive control whenever possible to demonstrate assay sensitivity that should be expected from a known response.

During the meeting, attention was focused on 3 specific areas that would have greatest impact on the ability to judge the validity of conclusions made in published work studying pain and analgesia: reduction of publication bias, increased ability to replicate the findings of others, and increased transparency of reporting of data. Preclinical research that reaches the level sufficient for publication (ie, is not merely a “pilot” experiment) can be believed of as existing in 2 distinct modes (Fig. 1): (1) exploratory or “hypothesis-generating” research that aims to generate theories pertaining to the pathophysiology of disease and (2) confirmatory or “hypothesis-testing” research that aims to demonstrate reproducible treatment effects in relevant animal models. Absolute conformity in experimental design and execution of exploratory investigations across laboratories and geographical boundaries is extremely difficult to achieve, and spending time to reorganize one's environment to become optimized for full replication of other's work is probably not the best use of limited resources. Most believe that scientists designing and reporting exploratory and confirmatory studies should use similar (if not the same) standards of rigor and lack of bias, and this can be achieved by attending to some simple and yet critical standards. Acknowledging that a study was performed with such a set of standards would allow improved ability to interpret the results of studies, a faster and more efficient compilation of the results obtained in the pain field for meta-analyses, and also hopefully reduce the impact of publication bias.

### 3.1. Problem of publication bias

Publication bias is present when research is published that does not represent the total body of research conducted. In part, publication bias results from the pressure to publish positive studies in high-impact journals. Publication bias has been studied extensively and to some extent mitigated in the clinical literature (eg, by the requirement to register all trials in clinicaltrials.gov), although the reporting of results of those trials is still not guaranteed. Publication bias is less well studied in the basic science/preclinical literature. In an analysis of several preclinical studies in different fields, including stroke, Parkinson disease, multiple sclerosis, and amyotrophic lateral sclerosis, it was concluded that there was publication bias in many of these areas.^19^ Among the contributors to this bias was failure to publish “nonpositive” data. Nonpositive data can be divided into “negative data” (a statistically significant effect in the direction opposite from that hypothesized or from that already published) and what we have termed “neutral data.” In our use of the term neutral data, we include data that clearly show no statistically significant effect over a narrow or wide confidence interval. However, neutral data may also be described as “inconclusive data” assuming that the confidence interval for the treatment effect contains values that might be considered to be relevant.^1^ Failure to publish nonpositive data presents ethical concerns as the same experiments may be repeated unnecessarily in other laboratories. The lack of balance also leads to overtly optimistic claims or the generation of inaccurate hypotheses.^16^ Conversely, publication of negative or neutral findings based on poor experimental methodology could deter others attempting replication, leaving the field with a false-negative result; thus, there is a critical need for transparency in reporting all experimental details so that the quality of research might be assessed.

#### 3.1.1. Reducing publication bias

Ideally, the decision to publish an article should be primarily based on the overall importance of the hypothesis being tested and the rigor of experimental design, conduct, analysis, and reporting and less on the “positivity,” “neutrality,” or “negativity” of results—all 3 types of findings are important for the field to know, provided the studies are of high quality. However, in the hierarchy of “desirability to publish,” there is no question that exciting, positive data are often prioritized for publication over “negative” data largely irrespective of the quality of experimental design and conduct; “neutral” data are very low down the list, and it is difficult at present to see the culture changing. With the exception of a few (eg, *PLoS One*), journals rarely publish negative and neutral findings. Specifically to reduce publication bias, what is needed is the publication of negative/neutral data in opposition to previously published positive data (if they exist). In essence, this greatly expands the “peer-review” process. One possibility is that journals could make a section available for letters or short communications where negative and neutral data can be reported (with simple statements demonstrating attention to the internal validity of these studies, ie, randomization, blinding, sample sizes, concealed allocation, adequate sample size). To be more palatable to the journals, these reports could be considered outside the scope of impact factor calculation while still being eligible for citation and future reviews. The value of such publications would become clear in reviews where “positive” findings could be set against available peer-reviewed “nonpositive” data, giving the reader the opportunity to judge the validity of a particular hypothesis or study. In addition, it should be noted that funding agencies, at least in the United States, receive updates on the progress of projects and could simply request that a brief summary of experiments that did not produce positive results be included; such data could be posted in a database accessible to others. One specific database already exists for biomedical research awards and contracts provided by the Department of Defense (DoD). The DoD requires Principal Investigators to submit thorough reports containing detailed experimental methods, data, and explanations of any deviations from the original statement of work. Reports are scientifically reviewed and Principal Investigators are provided feedback if required. These reports are publicly available at the Defense Technical Information Center (www.dtic.mil). At the repository called “Figshare” (www.figshare.com), it is already possible to post data in the form of articles, figures, spreadsheets, and other formats that are shared publicly. Although postings within Figshare are not refereed, these data could prove useful to some investigators. These figures should be accompanied by a brief discussion written by the scientist accompanying any nonsignificant data, explaining possible reasons for lack of significance (eg, incorrect hypothesis, methodology not optimized, study underpowered). At present, all publicly available research on Figshare gets allocated a DataCite DOI so that the research can be cited. However, we also recognize that citing articles and results that have not undergone peer review raise additional concerns, and believe that the presentation of findings from these sources should be accompanied by appropriate qualifications. It was therefore recognized that journals and funders have an important role to play in controlling publication bias.

### 3.2. Problem of repeating the work of others

A major requirement of scientific experimentation is the ability to repeat the findings of others; in this way, observations move closer to fact. Repeating experiments takes time and involves use of resources that could be directed towards discovering new findings. Nevertheless, if a scientist builds replication into any study, then confidence in the findings should increase. Although important throughout science, the need for replication is critical before embarking on a drug discovery program. Failure to replicate certainly raises a worry as to the robustness of the reported findings but does not necessarily mean that the original publication was incorrect as there may be environmental variations and procedural factors beyond the control of the different institutions that limit the ability to exactly recreate experimental conditions that were described in the publication being followed. Transparent reporting of experimental procedures is therefore essential to enable others to repeat the experimental procedures as faithfully as possible. Transparent reporting will also facilitate the interpretation of study results and meta-analyses that aggregate across studies by making sure that low-quality studies can be accurately identified and excluded. Transparent reporting has been shown to be a serious problem with certain key components of internal validity—randomization, blinding, and power calculations—only being reported in a minority of publications sampled.^7,15^

#### 3.2.1. Making replication easier and more attractive

Recent publications from Bayer^3^ and Amgen^13^ reported that the percentage of published findings in high profile journals that could not be replicated internally was very high, at least in preclinical cancer drug development, and these publications provoked much discussion. Although the authors did not report their experimental procedures or data regarding which studies were not replicated, these 2 reports did bring to the surface the worryingly high number of targets that are evaluated by scientists in industry but dropped because of an inability to repeat published findings. We should strive to improve reporting standards to reduce this attrition.

One recent attempt was reported^8^ to improve the ability across laboratories to repeat experiments. The publication consisted of a very detailed set of reporting guidelines called ARRIVE (Animal Research: Reporting In Vivo Experiments) that listed 20 separate items in a checklist to consider when writing a scientific article (see https://www.nc3rs.org.uk/arrive-guidelines). At the time of publication, the authors noted that although there were reporting guidelines for clinical trials (CONSORT) and metabolomics and gene expression studies, guidelines for reporting in vivo experiments were lacking. Many journals now recommend using the ARRIVE guidelines when reporting in vivo experiments, but, as yet there is little association between endorsement of preclinical guidelines by journals and their general adoption by the community, even at the most impactful publications.^2^ Whether the adoption of rigorous reporting guidelines such as ARRIVE has a positive impact on experimental reporting standards and preclinical reproducibility remains to be determined.

In terms of making replication more attractive, scientists could be encouraged to repeat work through the use of multicenter consortia; although the overall cost would be higher, the cost to each individual group would be the same. As the consortium would be working together, methodological details would be shared and experiments faithfully performed in different laboratories according to standardized protocols. An example of this approach is already evident in Europe through the Innovative Medicines Initiative that funds Europain (http://www.imi.europa.eu/content/europain), consortia of academic and industrial scientists studying the effect of chronic pain on different biological domains. This consortium approach would seem to be a relatively efficient way to evaluate the robustness of a particular finding. Although this approach is clearly not possible for every new finding, collaboration is an option open to scientists in general. Industry often has the technical capacity to replicate, and ideally, this resource should be tapped for precompetitive studies in public–private partnerships.

### 3.3. Problem of data presentation (increasing transparency)

The most common way to present data from a continuous outcome variable is to show a figure, such as a bar chart or line graph, that illustrates the mean ± SD (or more commonly SEM). A *P* value is often calculated indicating the probability of observing group differences at least as extreme as that observed if there was no true group difference (eg, no effect of the intervention). However, “*P*” values are poor summaries of evidence, as they do not convey information regarding the magnitude of the effect, the variability of the responses, and the biological relevance of the findings. Transparent presentation of experimental data would enable others to better evaluate the significance of the findings.

#### 3.3.1. Clearer methods of data presentation

A better way to show data than simply showing the mean ± SEM may be to report all individual points in each experimental group. This approach gives the reader the best appreciation of the variability and distribution of the data. An excellent option for presentation of results is to provide confidence interval estimates of the parameters of interest (eg, difference in means between groups). The width of the confidence interval indicates the precision associated with the estimate. A very wide interval could indicate that more data are required before a useful conclusion can be drawn as to the magnitude of the effect, if any. In the case of a difference in means between groups, the confidence interval would fail to include the value of zero if, and only if, the group difference is statistically significant. This duality between confidence interval estimation and hypothesis testing is useful for interpretation of the results.

## 4. Reducing experimental bias and misinterpretation of results by enhancing internal validity

There is a concern that the biological significance of many findings in the preclinical arena is exaggerated and a typical concluding sentence similar to “the findings of this study demonstrate that X may be a novel treatment for pain or a target for novel analgesics,” although absolutely not unique to pain research findings, is often not adequately supported by the data presented but may help to get the article published in a high impact journal. To counter this, reducing experimental bias by enhancing internal validity should lead to more robust, reproducible observations and should therefore be routinely considered when designing preclinical experiments (Table 1).

**Table 1:**
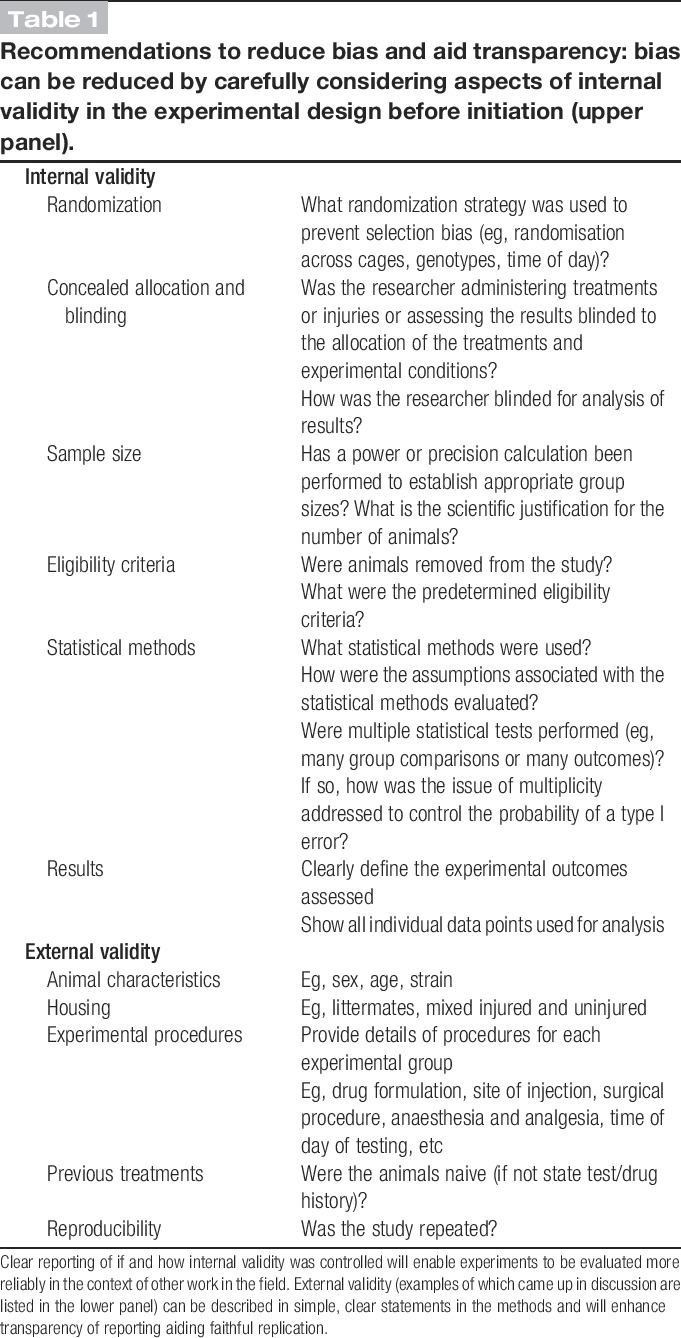
Recommendations to reduce bias and aid transparency: bias can be reduced by carefully considering aspects of internal validity in the experimental design before initiation (upper panel).

As alluded to in the Introduction, a number of aspects of study design that influence internal validity, if not given sufficient attention, may, alone or in combination, lead to bias and incorrect interpretation of results.^12,15^ In some cases, it is possible that the effects of blinding and randomization may be small, but this does not mean that they should not be used wherever possible to reduce bias because there are many instances where these factors can influence effect sizes. A recent systematic review of publications reporting findings from the use of bone cancer pain models in animals underscored the importance of internal validity.^4^ The authors identified 831 unique records from three electronic databases (PubMed, ISI Web of Science, and Ovid Embase in July 2011) of which 150 satisfied their eligibility criteria. In common with other meta-analyses, only a third of the articles analyzed (from a total of 150) reported the use of blinding, 11% reported randomization, and none of the publications reported sample size calculations. Overall, the authors concluded that those studies reporting measures to reduce bias found smaller differences in behavioral outcomes between tumor-bearing and control animals. The findings in the study just described are very similar to those reported in Reference 14 in a small sample study of preclinical nerve injury studies. A number of key considerations that should be used to reduce bias are listed in Table 1 and discussed below.

### 4.1. Sample size

Sample size refers to the number of experimental units (eg, individual animal, cage of animals). Inadequate sample size is a primary reason for incorrect conclusions. A sample size that is too small runs the risk of failing to detect important effects; however, a sample size that is too large puts more animals at risk than necessary and is wasteful of resources. Sample size, therefore, is not only relevant to the possibility of incorrect interpretation of the findings but also raises ethical issues. One of the goals of the in vivo scientist is to use the minimum number of animals consistent with the scientific objective (the 3Rs principle of reduction), and this number can be determined using power calculations. Using too many or too few animals would be inconsistent with the 3Rs principle. This concern is also addressed by institutional review boards when reviewing clinical trials—the minimum number of humans should be exposed to the risk of the intervention to adequately test the hypothesis. In a recent study,^6^ 2095 citations were examined that reported 26 different sets of guidelines for the conduct of preclinical efficacy studies across several fields of science. These 26 sets of guidelines contained 55 distinct recommendations. The authors found that 23 of the 26 sets of guidelines stated the need for power calculations. However, despite recognition of the importance of power calculations, the reporting of their use is routinely less than 10% in articles sampled.^14,16^

In the pain field, across a broad range of assays, the median sample size seems to be approximately n = 9 rats or mice per group.^11^ However, because of the lack of reporting of power calculations to determine group sample sizes, it is not clear whether the sample sizes are due to convention or to formal consideration of the power of the study. There is no magic number for group sample size because different study designs, including different outcome variables, yield different sample size requirements. The number of animals in an experiment, like the number of humans in a clinical trial, should be determined by a process: specification of a scientific hypothesis, translation into a statistical hypothesis, and specification of other design elements such as the primary outcome variable, the primary statistical test to be used, the significance level for the test, the desired power, the variance of the outcome variable (for a continuous outcome variable), and the group difference to be detected (effect size). Specification of the latter 2 quantities (ie, variance and effect size) can present challenges in preclinical studies, particularly if novel phenomena are being investigated. Pilot studies can be helpful in this regard before undertaking a confirmatory experiment. Indeed, it is stated in the U.S. Guide for the Care and Use of Laboratory Animals that “If little is known about a specific procedure, limited pilot studies, designed to assess both the procedure's effects on the animals and the skills of the research team and conducted under IACUC oversight, are appropriate” (p. 26). In addition, scientists should consider determining the size of an effect (group difference) that they would need to observe to be convinced that the treatment or intervention is worth investigating further.

Transparency in how a sample size was derived can also help provide context for interpretation in the face of multiple significance tests. Suppose that, during the course of an experiment, a test was conducted on a small number of mice and the result was not significant at a significance level of 5%. The investigator then added mice to the experiment and repeated the test at the 5% significance level, this time obtaining a significant result. The result of the final test is then reported in the literature. The false-positive error rate for this experiment, however, is greater than 5% given that multiple tests were conducted. The use of a more stringent significance level to account for multiple testing and the sequential nature of the experiment would be indicated in this case.

A formal sample size calculation can be recommended for confirmatory hypothesis testing studies. The role of such a calculation in exploratory hypothesis-generating studies is less clear. We recommend that authors of the reports of preclinical research provide transparency as to whether the study is exploratory or confirmatory, and if a power calculation was not performed, to explicitly state this in the Methods section. The total number of significance tests performed should also be reported to provide multiplicity context (section 4.6).

This particular topic produced by far the most discussion with opposing views being extensively discussed on the merits of the need for such formal determination of sample size. Overall, most meeting participants believed that preclinical studies that tested a statistical hypothesis should rely on formal determination of sample size rather than on convention. Without a sufficient number of animals per experimental group, statistical power is reduced and the interpretability of the results is compromised. As standards of reporting increase, meta-analyses may help determine adequate group sizes for common endpoints used to measure pain in animals.

### 4.2. Concealed allocation

Concealed allocation refers to the practice of concealing the group or treatment assignment (ie, the allocation) of each experimental subject from the investigator (eg, the surgeon) until the moment of assignment. By shielding the investigator from knowing the group to which the next animal will be allocated, researchers are prevented from influencing which subjects are assigned to a given intervention group. Concealed allocation is different from randomization, which protects against selection bias by balancing all confounding factors across the groups. It should also not be confused with blinding, which protects against observer bias after allocation.

Concealed allocation is a critical component of good experimental design in clinical studies. Meta-analyses of human clinical studies estimate that the efficacy of an intervention is exaggerated by 30% to 40% in studies with unclear or inadequately concealed assignments.^17,18^ However, in preclinical studies, it is rarely considered, and many of the participants in the PPRECISE meeting were unaware of this principle. Applied to preclinical studies, examples where lack of concealed allocation could introduce bias include (1) an investigator unconsciously (or otherwise) picking healthier subjects for the intervention group or (2) the practice of assigning an animal to the sham surgery vs nerve-injured groups' midsurgery based on how well the surgery is progressing and if the investigator easily identified the nerve. If the allocations are done in advance and revealed only at the moment of treatment or after the nerve is exposed, then this bias can be mitigated. There was an overall consensus that the use or not of concealed allocation should be reported.

### 4.3. Blinding (masking)

This is absolutely crucial at all levels of experimentation, from allocation of groups, testing of responses, to final analysis of data. However, it is recognized that it is not always possible to be truly blind to certain test conditions (eg, an inflamed hind paw or the adopted position of the foot after a peripheral nerve injury), and this should be reported in publications. Measures that reduce the impact of such loss of blinding (eg, automation or video recording where possible or the inclusion of a dummy group in an otherwise 2-group comparative study) should always be considered to reduce bias. There was a consensus that the use or not of blinding should be reported.

### 4.4. Randomization

Randomization is the foundation for statistical inference. It ensures the expectation of balance among the comparison groups with respect to the distributions of all potentially confounding factors regardless of whether you know about them or whether you have measured them. It is therefore very powerful. Randomization of treatment and testing order avoids confounds, such as time of day, cage or litter effects, and subconscious influencing of the tester. Randomization can be performed simply by entering all information of animals into an Excel spreadsheet and assigning each one a random number using the “rand()” function and then sorting them into ascending or descending order, which results in a randomized arrangement of treatments (see http://www.3rs-reduction.co.uk/html/5__avoiding_bias.html). There was a consensus that the use or not of randomization should be reported.

### 4.5. Eligibility criteria

Predetermined eligibility criteria are scientifically and ethically important considerations and must be reported to aid transparency and to account for each animal entered into an experiment. The criteria should always be set before the experiment begins, and a simple statement in the methods can explain such inclusion criteria and any exclusion criteria. For example, it is acceptable to exclude animals that have been tested for baseline responses before entering them into the test phase if they do not respond within experimentally predetermined norms. Or, if an animal is found to be ill after, but not related to, treatment with a particular compound, eg, poorly performed injection, it may and indeed should be excluded from testing. However, it is very important to be sure that exclusion is independent of treatment assignment to avoid compromising the integrity provided by randomization. When dealing with “outliers” in the outcome data, some at the meeting said that they used “the 3–standard deviation rule”; that is exclusion of data lying more than 3 SDs away from the group mean. However, bias cannot be avoided when using such rules unless there is a good understanding of why the outliers occurred. Although some at the workshop believed that all data should be included, there was a consensus that if data were excluded, it must be reported.

### 4.6. Multiplicity

Issues of multiple statistical testing are pervasive in almost all preclinical research. These typically manifest as inclusion of multiple comparison groups, multiple outcome variables, multiple time points at which outcomes are measured, multiple methods for statistical analysis (eg, including and excluding “outliers” or different methods for dealing with missing data), multiple secondary analyses (eg, subgroup analyses), and interim analyses of accumulating data. The implications of multiple statistical testing are well known: if each of a collection of statistical tests is performed using a significance level of, say, 5%, the probability that at least one of the results is statistically significant when the null hypothesis is actually true is greater than 5%, sometimes substantially greater. There is an extensive literature on the problem of multiplicity and ways to address it, although the subject is somewhat contentious as there are several perspectives on how to approach the problem.^17,18^

The multiplicity issue is arguably less critical in exploratory research, in which the researcher may be willing to accept a higher probability of a false-positive result (ie, type I error) knowing that it will have to be confirmed in subsequent research (Figure 1), but if the experimental data are published before replication is confirmed, this is a clear problem (Section 3.1, “publication bias”). For confirmatory studies, however, researchers must acknowledge the problem of multiplicity and, ideally, address it at the design stage. One way to do this is to provide clear prioritization of comparisons, outcomes, analyses, and, so forth, as one normally does in confirmatory clinical trials in human subjects. Such prioritization should be clearly documented in advance of the experiment and fully disclosed at the stage of publication to ensure proper interpretation of the study results. Another approach is to use appropriate statistical methods to adjust for multiple testing. Overall, transparent reporting of all statistical testing that was performed is recommended to allow readers judge whether the multiplicity issues have been addressed appropriately.

## 5. Discussion

The reporting of experimental methods and data in the field of pain research must become more transparent, and greater attention needs to be paid to internal validity (Table 1) to improve the rigor of experimental design, which will increase the interpretability of studies and enable high-quality meta-analyses. We believe that these improvements will have a major influence in ensuring the future success of pain research. Without doubt, some of the topics discussed at the meeting produced far more debate than others and of course this reflected the level to which there was agreement on how important that issue was perceived to be to the impact on bias or transparency of reporting.

In particular, this meeting identified major disagreements among experts in the field with respect to the need for a priori power calculations (section 4.1) to define sample sizes. One of the major sticking points was how does one determine a meaningful effect size for something entirely novel. One way to view this would be to only consider large effect sizes, however although this would undoubtedly reduce the number of publications, not considering small effect sizes may be an oversimplification of the subject.

While acknowledging the unique status of pure discovery (“hypothesis-generating”) biology, it is fair to suggest that the vast majority of the literature does not belong to that category of research because many studies build on those initial breakthrough discoveries. However, if a study is using methods that are really so original that a sample size calculation cannot be performed, then the results should be acknowledged as “exploratory observations” rather than “findings.” Notwithstanding the issue of purely novel discovery biology, even if a completely novel pain mechanism is being evaluated, it is often the case that a well-established model and outcome measure are used for the in vivo experiments. In that case, it is possible to determine sample size by reference to effect sizes that have been seen to be reasonable in previous reports using the same model and outcome measure. Finally, if a trio of novel mechanism, novel model, and novel outcome measure is used in concert, then a suggested solution would be to have a comparative arm in the study where animals were treated with a known standard, for example, gabapentin (neuropathic pain), morphine (nociceptive pain), or ibuprofen (inflammatory pain) in a known model and with a known outcome measure. This would enable the author to compare the effect size in the novel scenario with that using a “standard” approach potentially highlighting the value of the new approach.

It will take more time, effort, and personnel to produce experimental studies with high-quality design and execution but lower-quality studies, while undoubtedly easier to perform, produce output that is of less use, filling the field with “information noise.” It can therefore be argued that lower-quality studies compromise the efficiency and timeliness with which the field as a whole moves forward. Journal editors, reviewers, and grant funders should encourage/require greater transparency of reporting, and many are beginning to do so. Universities have a duty to teach students the important principles of experimental design, sound data analysis, and transparent reporting from the start of their scientific education so that regardless of whether they continue in academic research or move to industry, publishing or funding bodies, they will adopt sound principals and be more critical of the published literature. Ultimately, this will lead to the issues discussed herein being greatly reduced.

These recommendations are relatively straightforward and overall benefits should be evident but will need to be assessed in an ongoing way (eg, see Reference 2). Other issues such as confirmation of experimental findings across independent groups and the publication of “negative” (and “neutral”) data will require a greater commitment by all stakeholders, but here again funders and journals must take the lead. Publication decisions should give greater weight to the rigor of the experimental design and conduct and less to the apparent novelty or “positive” nature of experimental findings. We believe that to achieve the goal of finding effective new treatments for patients suffering from pain, we need to confront and deal with these challenging issues. Conducting high-quality science that is fully reported does not preclude novelty and innovation in preclinical pain research; indeed, we would argue that it will facilitate it.

Since formulating our manuscript, we note that the National Institute of Neurological Disorders and Stroke (NINDS) has issued guidance to researchers which are, in the main, concordant with our suggestions (http://www.ninds.nih.gov/funding/transparency_in_reporting_guidance.pdf).

## Conflict of interest statement

The authors have no conflicts of interest to declare.

N. A. Andrews and A. Latrémolière have contributed equally to this article.

The findings, conclusions, and recommendations in this article are solely those of the authors, none of whom have financial conflicts of interest specifically related to the issues discussed in this article. At the time of the consensus meeting on which this article is based, 3 authors were employed by pharmaceutical companies and others had received consulting fees or honoraria from one or more pharmaceutical or device companies. Authors of this article who were not employed by industry or government at the time of the consensus meeting received travel stipends, hotel accommodations, and meals during the meeting from the Analgesic, Anesthetic, and Addiction Clinical Trial Translations, Innovations, Opportunities, and Networks (ACTTION) public–private partnership. No official endorsement by the Food and Drug Administration (FDA) or the pharmaceutical and device companies that have provided unrestricted grants to support the activities of ACTTION should be inferred. Financial support for this project was provided by ACTTION, which has received research contracts, grants, or other revenue from the FDA, multiple pharmaceutical and device companies, and other sources.
